# Anaerobic crystallization of proteins

**DOI:** 10.1007/s12551-017-0345-8

**Published:** 2017-12-02

**Authors:** Miki Senda, Toshiya Senda

**Affiliations:** Structural Biology Research Center, Photon Factory, Institute of Materials Structure Science, High Energy Accelerator Research Organization, 1-1 Oho, Tsukuba, Ibaraki 305-0801 Japan

**Keywords:** X-ray crystallography, Crystallization, Anaerobic conditions, Oxidation, Disulfide bridge, Reproducibility

## Abstract

Crystallization has been a bottleneck in the X-ray crystallography of proteins. Although many techniques have been developed to overcome this obstacle, the impurities caused by chemical reactions during crystallization have not been sufficiently considered. Oxidation of proteins, which can lead to poor reproducibility of the crystallization, is a prominent example. Protein oxidization in the crystallization droplet causes inter-molecular disulfide bridge formation, formation of oxidation film, and precipitation of proteins. These changes by oxidation are typically irreversible. The best approach for preventing protein oxidization during crystallization is anaerobic crystallization. Here we review the anaerobic crystallization of proteins, which was originally developed to trap a reaction intermediate of the enzyme in the crystal. We also summarize representative anaerobic crystallizations from our laboratory and the general setup of anaerobic crystallization.

## Introduction

Since the tertiary structure of proteins (or biological macromolecules) is a key to analyzing the molecular mechanisms underlying cellular biological processes, much research effort has been expended in analyzing tertiary structures. As a result, more than 130,000 coordinates have been deposited to date in the Protein Data Bank (Burley et al. 2017). While the use of single particle analysis by cryo-electron microscopy has expanded rapidly in recent years (Fernandez-Leiro and Scheres 2016), X-ray crystallography remains a major tool to determine the tertiary structures of proteins. One of the main advantages of X-ray crystallography is its high-throughput nature. The combination of a synchrotron light source, robotics, a pixel array type X-ray detector, and sophisticated software enable more than 100 diffraction data sets to be collected easily within 1 day. Once all these procedures are optimized, it should be possible to collect more than 500 data sets in that same time period. However, to achieve a high-throughput analysis, the biological macromolecule of interest needs to be purified and crystallized—and this step constitutes a bottleneck. To date, many techniques have been developed to overcome this bottleneck. For example, the sparse matrix method has greatly improved the success rate of the initial crystallization screening (Jancarik and Kim 1991). As a result, many screening kits are now commercially available. In addition, robotics has been introduced as a means to improve the efficiency and accuracy of the initial crystallization screening. Indeed, several crystallization robots are commercially available, including a few large-scale crystallization robots for protein crystallization (Morris et al. 1989). Since robots can handle small amounts of sample with high accuracy, their utilization is effective for realizing experiments with high reproducibility. In our laboratory, a large-scale, fully automated crystallization robot is in operation, which can perform every process of the crystallization as well as observe the crystallization droplets (Hiraki et al. 2006).

In addition to the crystallization methods themselves, new methods for the expression and purification of proteins have also accelerated the process of protein crystallization. Both bacterial expression systems and systems using eukaryotic cells have been developed, thereby expanding our targets for structural analysis. The combination of a high-yield expression system and a tag-purification method has improved the quality of purified recombinant proteins. Moreover, although the reconstitution of large protein complexes was previously considered impossible, it has become possible to reconstitute large protein complexes by using recombinant DNA techniques (Imasaki et al. 2011; Bieniossek et al. 2013; Nozawa et al. 2017).

In addition to these developments, assessment of the physicochemical purity, or physicochemical uniformity, has become a common procedure in recent years because conformational variety and thermodynamic instability are not advantageous for achieving crystallization. The physicochemical purity of samples has been pursued via several methods, of which the most frequently used is the truncation of intrinsically disordered regions of the target protein (Chait 1994). In the case of multi-domain proteins, isolation of a single domain is an effective method for crystallization. Since the relative arrangement of the domains can easily be changed in the multi-domain protein, a reasonable strategy is to isolate a domain for crystallization. In addition, the thermodynamic stability of the protein is often assessed before crystallization using the differential scanning fluorimetry (DSF) method (Ericsson et al. 2006), which can quickly estimate the melting temperature (*T*m). One of the striking examples of using thermo-stabilization in protein crystallization is the introduction of a T4-lysozyme in a loop region of the G-protein-coupled receptors prior to their crystallization (Cherezov et al. 2007). Many membrane proteins have also been crystallized via thermo-stabilization (Hattori et al. 2012). On the basis of these results, the DSF method has become a popular approach for analyzing the stability of proteins. Indeed, the optimization of buffer conditions using DSF has proven effective for crystallization. Size exclusion chromatography is also routinely utilized to quickly assess the physicochemical properties of a purified protein sample, such as its aggregation and physicochemical heterogeneity.

While these advanced techniques have significantly improved the success rate of protein crystallization, several problems remain. For example, chemical changes of the protein in the crystallization droplet can lead to crystallization failure. A well-known example of just such a chemical change during crystallization is oxidation of the protein sample, which causes inter-molecular disulfide bridge formation, formation of oxidation film, and precipitation of proteins. In our experience, the success rate of the crystallization can be improved by avoiding oxidation of the crystallization sample. In this review, therefore, we provide a brief history of the anaerobic crystallization method used to trap a reaction intermediate and describe the possible advantages of this method, as well as summarizing our experiences with this technique.

## Anaerobic crystallization to isolate reaction intermediates

Anaerobic conditions in protein crystallography have been utilized to isolate reaction intermediates of enzymes with a redox center. The redox center of the enzyme controls the reactivity of the enzyme by changing its redox state. When the reduced state is a reactive form of the enzyme, structural information of the reduced form should be obtained to explain the catalytic reaction mechanism. However, in many cases, the reduced form is unstable under aerobic conditions due to its oxidation by oxygen molecules. Thus, to trap the reduced form of the enzyme in the crystal, the enzyme should be crystallized under anaerobic conditions. It would also be possible to reduce a crystal of the oxidized form of the enzyme by soaking the crystal in a solution supplemented with a reducing reagent under anaerobic conditions, unless the crystal is broken by the reduction of the enzyme.

One of the first examples of anaerobic crystallization was that of isopenicillin *N*-synthase by the Baldwin group. These researchers crystallized a substrate complex form of the isopenicillin *N*-synthase under anaerobic conditions, revealing the crystal structure of its reaction intermediate (Roach et al. 1996, 1997). Following this advance, many other crystal structures of reaction intermediates that were prepared under anaerobic conditions were reported, including flavoproteins (Senda et al. 2007a), proteins with an iron-sulfur cluster (Colbert et al. 2000; Hsieh et al. 2005; Senda et al. 2007a), and non-heme iron proteins (Uragami et al. 2001; Vaillancourt et al. 2002; Sato et al. 2002). A trapped reaction intermediate can also be utilized to obtain the following reaction intermediate of the catalytic reaction. In the case of naphthalene dioxygenase, an oxygen molecule was introduced into a frozen crystal of a reaction intermediate, yielding an O_2_-binding form of this enzyme (Karlsson et al. 2003). Several similar examples have also been reported (Tocheva et al. 2004; Kovaleva and Lipscomb 2007). A redox-dependent complex was also crystallized under anaerobic conditions as follows. Ferredoxin and ferredoxin reductase interact in a redox-dependent manner. Upon the reduction of flavin adenine dinucleotide (FAD), BphA4, an FAD-containing ferredoxin reductase, increases its affinity for ferredoxin BphA3. The BphA3–BphA4 complex can then be isolated under anaerobic conditions (Senda et al. 2007b), as can the crystals of this redox-dependent BphA3–BphA4 complex (Senda et al. 2007c).

## Anaerobic crystallization to avoid oxidation

Another advantage of anaerobic crystallization is that chemical purity can be maintained by avoiding oxidation of the target protein and crystallization reagents. When free Cys residues are present in the target protein, these residues can form an intra/inter-molecular disulfide bridge(s), causing heterogeneity of the protein in solution. This obstruction of crystallization by oxidation is a well-established phenomenon. The addition of a reducing reagent is a typical strategy to avoid the random formation of the intra/inter-molecular disulfide bridges. However, since it is rather difficult to completely avoid oxidation of the sample for a long period under aerobic conditions, the reproducibility of the experiment tends to be poor. Furthermore, oxidization of the reducing reagent results in heterogeneity of the crystallization solution, which would cause problems with the crystallization. It is therefore quite difficult to maintain all the conditions the same across many crystallization trials using a reducing reagent. To avoid heterogeneity in the crystallization solution, the use of anaerobic conditions is a potent method. For example, anaerobic crystallization can easily avoid the formation of an inter-molecular disulfide bridge and increase the reproducibility of crystallization, as observed in the crystallization of oxidized and reduced forms of BphA3 (Senda et al. 2007b).

**Fig. 1:**
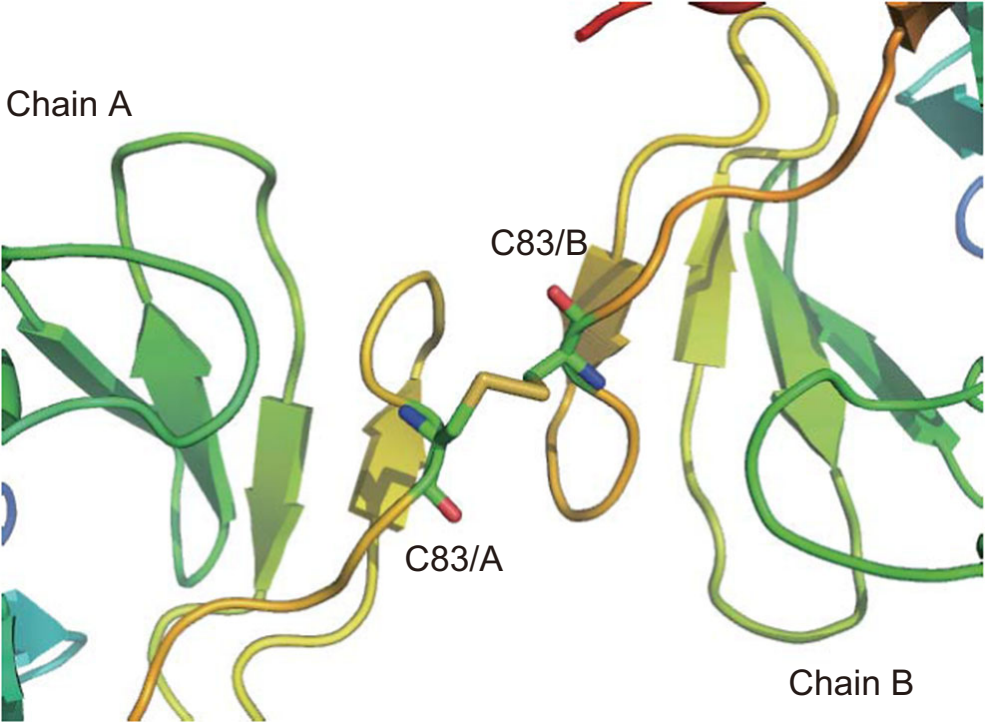
Disulfide bridge connecting two BphA3 molecules. BphA3 is a monomeric Rieske-type [2Fe-2S] ferredoxin. However, a disulfide bridge was formed between two BphA3 molecules in an aerobic crystallization (Senda et al. 2006)

BphA3 is a ferredoxin that contains a [2Fe-2S] cluster. We determined the crystal structures of the oxidized and reduced forms of BphA3 to explain its redox-dependent interaction with ferredoxin reductase BphA4. Initially, we crystallized the oxidized form of BphA3 (Senda et al. 2006). Since the oxidized form of BphA3 [BphA3(ox)] can be maintained under aerobic conditions, we performed the crystallization of BphA3(ox) under aerobic conditions. However, the reproducibility of the BphA3(ox) crystallization was very poor, and only a few crystals were obtained despite repeated efforts. The crystal structure of BphA3(ox) demonstrated that BphA3(ox) forms a dimer via an intermolecular disulfide bridge; that is, a free Cys residue in BphA3 formed an intermolecular disulfide bridge during the crystallization, which in turn formed a disulfide bridge-mediated dimer (Senda et al. 2007a) (Fig. 1). The formation of the inter-molecular disulfide bridge occurs randomly in the crystallization solution, and thus it is hard to control the oxidation. There are more than two Cys residues in BphA3, causing heterogeneity of inter-molecular disulfide bond formation. This heterogeneity is likely to lead to poor reproducibility of the crystallization under aerobic conditions. On the other hand, the crystallization of BphA3 in the reduced form [BphA3(rd)] showed quite good reproducibility (Senda et al. 2007b). To avoid the oxidation of the reduced [2Fe-2S] cluster in BphA3(rd), we carried out all crystallization procedures under anaerobic conditions. The anaerobic conditions avoided the oxidization of not only the [2Fe-2S] cluster but also of the free Cys residue in BphA3(rd). As a result, the chemical purity of the protein sample was preserved, leading to the high reproducibility of the crystallization experiments (Senda et al. 2007b).

## General setup for anaerobic crystallization

**Fig. 2:**
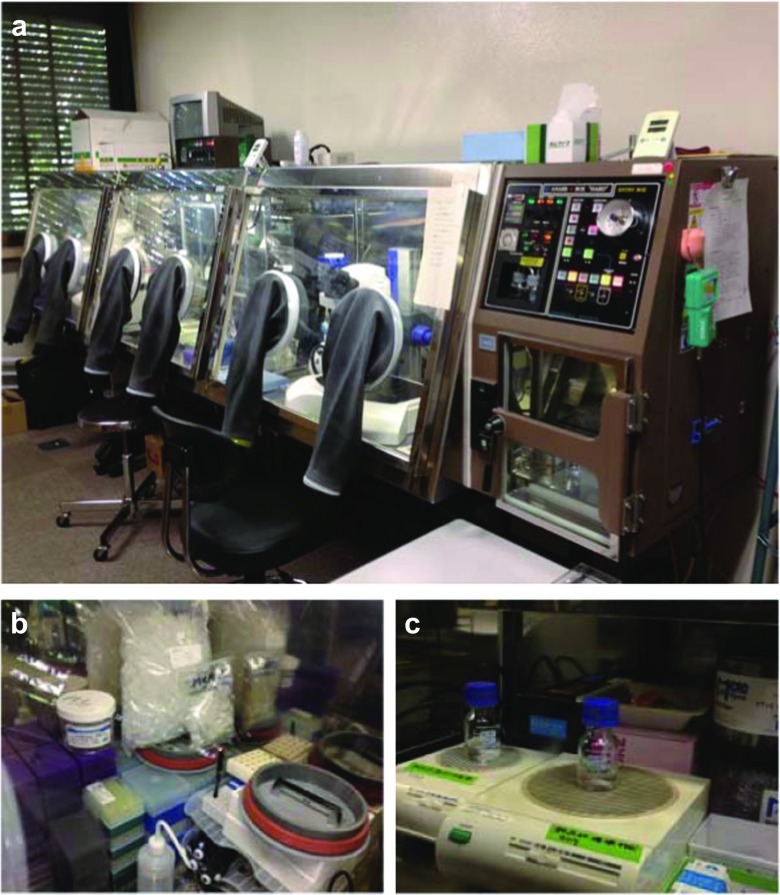
Setup for anaerobic crystallization. **a** Anaerobic chamber specifically modified for anaerobic crystallization (model Anaerobox 'HARD' ; Hirasawa, Tokyo, Japan), with three separate ‘rooms’ and containing a microscope for crystal observation. **b** Plasticware stored in the anaerobic chamber. **c** Magnetic stirrers in anaerobic chamber for degassing of buffers

As described in the preceding section, anaerobic crystallization is an effective process for preserving the chemical purity of the protein sample. In this section, we describe a general setup for anaerobic crystallization. First, an anaerobic chamber is needed (Fig. 2a), and several types are commercially available. Typically, the chamber is filled with a gas mixture of N_2_ and H_2_. The anaerobic conditions inside the chamber can be maintained by an oxygen-consuming reaction on a Pd catalyst. On the catalyst, O_2_ molecules in the chamber react with H_2_ molecules in the gas mixture to form H_2_O molecules, thereby reducing the oxygen level in the chamber. To keep the oxygen level low, the gas mixture should be fresh all the time. Therefore, it is highly recommended that the program for introducing the gas mixture into the anaerobic chamber be optimized. In our setup, a gas mixture (96% N_2_, 4% H_2_) is introduced into the anaerobic chamber for 1 min every hour. Prior to starting the experiments, it is highly recommended that the O_2_ level be checked using an anaerobic indicator, such as an Oxoid anaerobic indicator (Thermo Fisher Scientific, Boston, MA), unless the chamber is equipped with an oxygen monitor.

**Fig. 3:**
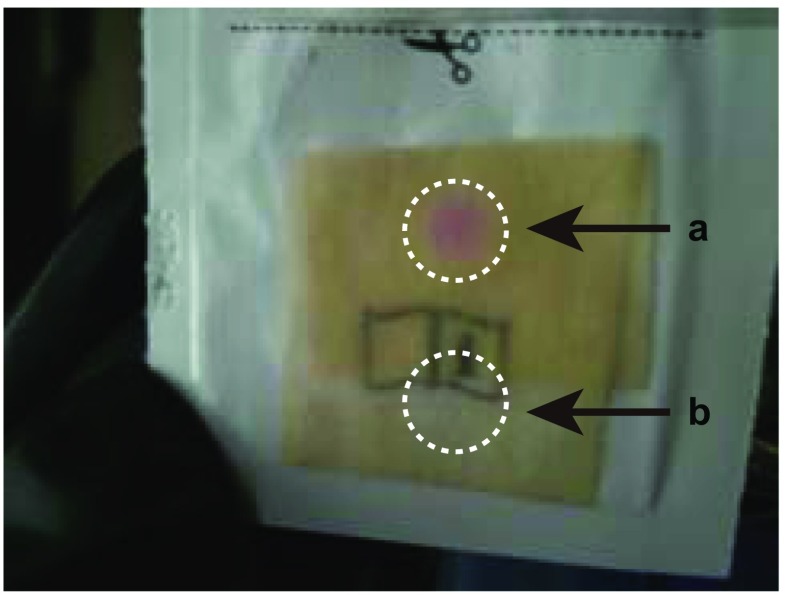
Checking the oxygen level with an anaerobic indicator. The pink spot indicated by the arrow marked* a* shows that the oxygen level of the buffer is not sufficient for an anaerobic experiment. The sample indicated by the arrow labeled* b* shows that the oxygen level of the buffer is sufficient for the anaerobic experiment

To maintain the anaerobic conditions throughout the experiments, it is not sufficient to optimize the program for introducing the gas mixture, but it is also crucial to remove the O_2_ molecules from any plasticware, sample solution, or buffer solution used in the experiment. In particular, the removal of O_2_ molecules from any plasticware to be used in the anaerobic chamber must be done thoroughly and carefully. Since O_2_ molecules are hydrophobic in nature, they can be easily adsorbed onto the surface of plasticware. In our laboratory, plasticware intended for anaerobic use is usually kept in the anaerobic chamber for more than 2 weeks before use in order to allow the O_2_ molecules on the plastic surfaces to be exchanged with N_2_ (Fig. 2b). Protein and crystallization solutions should also be treated under anaerobic conditions to remove O_2_ molecules (Fig. 2c). Stirring a solution under anaerobic conditions can accelerate the removal of oxygen molecules; to monitor the removal progress, the O_2_ level of these solutions can be checked with an anaerobic indicator (Fig. 3).

For convenient anaerobic crystallization, it is preferable to equip the chamber with an incubator and a microscope. By sealing the crystallization plate completely with vacuum grease, such as Apiezon grease (Apiezon, Manchester UK), it becomes possible to remove the crystallization plate from the anaerobic chamber and keep it in an aerobic incubator. However, it is awkward and inconvenient to handle a crystallization plate sealed with vacuum grease. Therefore, it is preferable to perform the incubation and observation of the crystallization plates in the anaerobic chamber. Since a microscope can easily be equipped with a CCD camera, images of crystallization droplets can be observed through a monitor inside or outside the chamber. If the microscope is in the anaerobic chamber, it is possible to freeze the crystal inside the anaerobic chamber.

## Effects of anaerobic crystallization

**Fig. 4:**
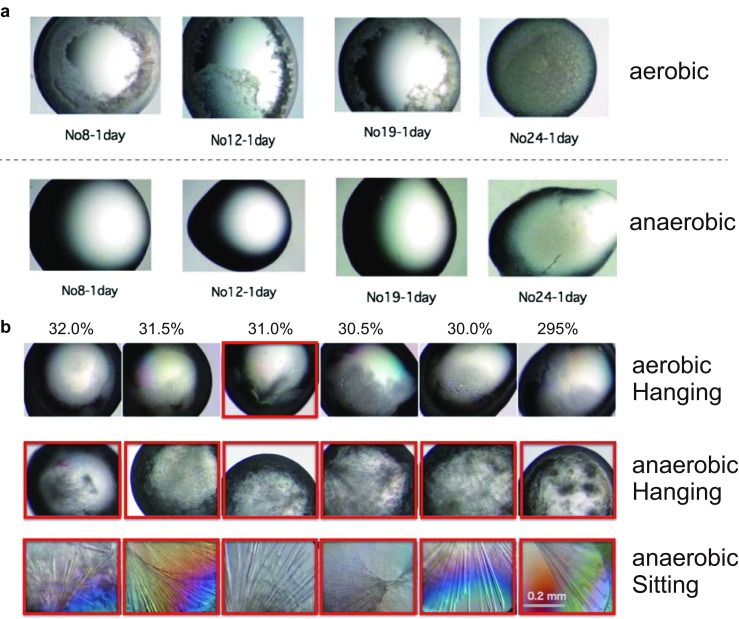
Comparison of aerobic and anaerobic crystallization. **a** Difference between aerobic and anaerobic crystallization in the initial screening of the tandem SH2 (Src homology 2) domain of the tyrosine phosphatase SHP2. Protein precipitation frequently occurred under aerobic conditions, suggesting that oxidation of the protein sample caused protein precipitation. **b** Differences between aerobic and anaerobic crystallization of the tandem SH2 domain in complex with a phosphorylated EPIYA-C peptide. While crystallization under aerobic conditions rarely produced crystals, crystallizations under anaerobic conditions showed good reproducibility

To analyze the effects of anaerobic crystallization, we compared the results of the initial anaerobic screening with those of aerobic screening using the tandem SH2 (Src homology 2) domain of the tyrosine phosphatase SHP2 in complex with a phosphorylated peptide (Hayashi et al. 2017). The phosphorylated peptide is part of the oncoprotein CagA, which is derived from *Helicobacter pylori*. The interaction between the tandem SH2 domain and the phosphorylated CagA peptide disturbs cellular signaling, causing tumorigenesis (Ohnishi et al. 2008). To compare the aerobic and anaerobic crystallization, we took a photograph of each crystallization droplet after 30 s and again after 1 day of crystallization. While no significant differences were observed between the aerobic and anaerobic conditions after 30 s of crystallization, there were clear differences between the two sets of conditions after 1 day (Fig. 4a). Several droplets under the aerobic conditions showed heavy protein precipitation, which seemed to hamper the crystallization of the protein. Under anaerobic conditions, however, some of the corresponding droplets continued to be clear. This result suggested that the heavy precipitation observed under aerobic conditions is due to the oxidization of proteins and/or crystallization reagents in the crystallization droplet and that crystallization under anaerobic conditions could prevent the formation of heavy precipitants. Therefore, to avoid unwanted oxidations of proteins and/or crystallization reagents, we performed subsequent crystallization trials of the tandem SH2 domain under anaerobic conditions.

Several trials of crystallization screening of the tandem SH2 domain with various phosphorylated peptides were performed under anaerobic conditions, and eventually micro-crystals of the tandem SH2 domain in complex with the phosphorylated EPIYA-C peptide were obtained. After optimization of the crystallization conditions, rod-shaped crystals were obtained in a reproducible manner under anaerobic conditions in the sitting drop setting (Fig. 4b). Interestingly, the same crystallization conditions rarely gave crystals under aerobic conditions; precipitation occurred in most of these trials (Fig. 4b), suggesting that oxidation of the protein and/or crystallization reagents has adverse effects on crystallization. In many cases, oxidation of the proteins and/or crystallization reagents seem to cause precipitation, which reduces the concentration of protein in the droplet and hampers protein crystallization. Since oxidized protein molecules can be regarded as impurities of the protein solution, oxidized protein molecules hamper protein crystallization. Moreover, in cases in which an oxidation film would be formed on the surface of the crystallization droplet under aerobic conditions, anaerobic crystallization will still be effective because the oxidation film will not form on the droplet surface under anaerobic conditions. Since formation of the oxidation film is typically irreversible and would reduce the concentration of the protein in the droplet, the oxidation film impairs the reproducibility of the crystallization experiment.

## Conclusion

While the oxidation of proteins and crystallization reagents has been recognized for some time, there has been no systematic search for fundamental solutions to the problem. Here, we have reviewed the topic of anaerobic crystallization and described how it frequently improves the results and reproducibility of crystallization experiments. Since the oxidation of the protein in the crystallization droplet causes a reduction in the purity of the protein, avoiding oxidation under the anaerobic conditions is theoretically reasonable.
